# Social cognitive impacts of digital art in metaverse environments: A cross-sectional study of user perception and behavioral patterns

**DOI:** 10.1371/journal.pone.0344776

**Published:** 2026-04-01

**Authors:** Lin Yang

**Affiliations:** Communication University of China Nanjing, Nanjing, Jiangsu, China; Guangdong University of Petrochemical Technology, CHINA

## Abstract

This cross-sectional study investigated the social cognitive correlates of digital art in metaverse environments through analysis of user perception and behavioral patterns. The research examined how exposure to digital art within immersive virtual spaces is associated with user cognition and identified demographic factors moderating these associations. Secondary analysis was conducted on multiple datasets including VREED (VR emotion dataset with physiological measures, n = 34), VR eye-tracking data (n = 152), virtual museum navigation records (~7 million), ArtEmis emotion attributions (455K annotations), and WikiArt metadata (80K artworks). Results revealed that digital art exposure showed significant positive associations with aesthetic perception and social value attribution, with moderate effect sizes. Three behavioral exploration patterns emerged: systematic explorers (31.6%), selective browsers (42.1%), and random wanderers (26.3%). Behavioral patterns partially mediated relationships between perception and social cognitive outcomes through emotional resonance, cognitive elaboration, and social mediation pathways. Significant moderation effects were identified for age (U-shaped pattern with strongest associations for 18–25 and 46 + groups), technology proficiency, and cultural background (differential patterns for collectivist versus individualist cultures). The findings demonstrate that digital art experiences in virtual environments are associated with meaningful differences in user perception and social cognition, though associations are moderate and heavily moderated by individual differences. This challenges assumptions about universal effects of metaverse technologies and highlights the need for adaptive, culturally-sensitive approaches to digital art presentation in virtual spaces.

## 1. Introduction

The emergence of the metaverse worlds has revolutionized the way people interact with virtual content, especially artistic presentation and consumption [1]. The three-dimensional interactive virtual worlds defined by consistent digital spaces where users interact through avatars and experience greater presence constitute a paradigm shift from the conventional two-dimensional online experiences [2]. As a post-reality universe merging physical reality with digital virtuality [3], virtual metaverse art covers a wide variety of forms from AI-created works to three-dimensional experiential installations, offering new creative possibilities and novel engagement opportunities for audiences [4]. Virtual environment architecture offers new kinds of spatial experience that transcend traditional artistic categories [5]. Technological advances in recent years have made metaverse more accessible through devices such as smartphones, expanding demographic access to virtual art worlds [6]. The worldwide metaverse economy, projected to achieve substantial economic value, has attracted significant investment in virtual worlds supporting cultural and artistic expression [7].

Virtual digital art research has developed rapidly, examining how immersive technology is employed to augment artistic experience and user interaction. Virtual reality art therapy demonstrates intricate cognitive processes within digital artistic activity, with various digital art modalities stimulating different neural pathways [8]. Virtual reality aesthetic experience is shaped by technological orientation, hedonic motivation, and personal traits, with varying emotional effects on spectators [9,10]. Immersive virtual reality in art education increases attention, enjoyment, and recall compared to conventional media [11]. Immersive virtual reality affects art education outcomes through its impact on flow state, cognitive load, brain state, and motivation [12]. Virtual aesthetics and the significance of artwork are remapped by digital technology within virtual environments, revolutionizing conventional curation practice and introducing new possibilities for artworks [13].

Social cognitive theory provides a robust foundation for explaining how virtual environments influence user behavior and perception. The theory’s focus on reciprocal determinism—the bidirectional process of continuous interaction between behavioral reactions, environmental influences, and personal factors—remains particularly relevant in virtual environments [14]. Observational learning and modeling function effectively in digital environments, with virtual social stimuli exerting powerful effects on user behavior and cognition [15]. The Cognitive-Affective-Social Theory of Learning in digital Environments extends conventional cognitive theories by incorporating social processes that affect individual learning and behavior in virtual environments [16]. Social presence and parasocial relationships in virtual environments influence user motivation, emotional reactions, and behavioral patterns [17]. Social learning theory persists as operational in contemporary digital learning environments [18,19].

User experience in metaverse environments reveals complex relationships between technological features, individual characteristics, and behavioral outcomes. Platform interoperability, usability, and social influence shape user perceptions through multiple interconnected factors [20]. Cross-sectional analyses of metaverse user behavior provide insights into demographic differences, usage patterns, and psychological impacts, with age-related differences in technology adoption and virtual environment engagement showing younger users demonstrating higher acceptance rates and different interaction patterns [21].

Despite growing interest in metaverse technologies and digital art, critical gaps remain in the current literature. Existing research primarily focuses on either technical capabilities or general user experiences, with insufficient attention to the psychological and social cognitive dimensions of digital art interaction in immersive environments. The moderating roles of demographic factors in shaping digital art experiences also remain largely unexplored. The intersection of social cognitive theory with metaverse-based digital art experiences represents an underexplored research domain with significant theoretical and practical implications. Current literature lacks comprehensive analyses that simultaneously examine both user perception patterns and behavioral responses to digital art in metaverse contexts.

**Fig 1:**
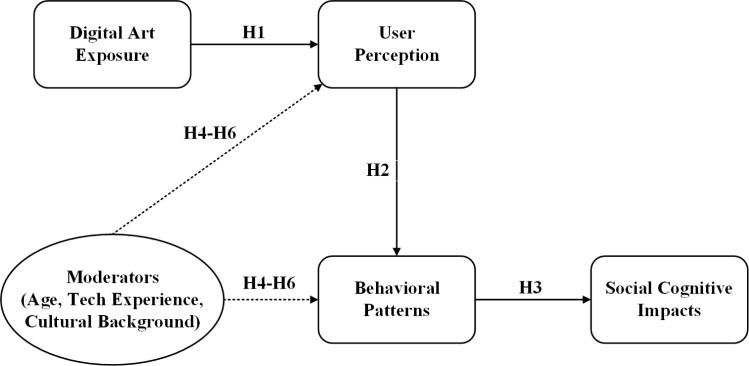
Conceptual model of research hypotheses.

The present study addresses these gaps by investigating the social cognitive correlates of digital art in metaverse environments. Given that capturing physiological responses, behavioral patterns, and self-reported perceptions within a single primary study would require extensive resources and specialized equipment, this research adopts a multi-dataset approach that integrates complementary secondary datasets to enable triangulation across measurement modalities. This study contributes to the literature in three ways: (1) applying social cognitive theory to metaverse-based digital art experiences, (2) examining perception-behavior relationships across physiological, behavioral, and self-report measures, and (3) investigating demographic moderators that have received limited attention in prior research. Based on social cognitive theory and existing research on virtual environments, this investigation proposes six hypotheses (Fig 1):

H1: Digital art exposure in metaverse environments is positively associated with user aesthetic perception and social value perception.

H2: User perception of digital art positively correlates with behavioral engagement patterns including consumption behaviors and social interaction patterns.

H3: Behavioral patterns mediate the relationship between digital art perception and broader social cognitive outcomes.

H4: Age moderates the relationship between digital art exposure and user perception/behavior patterns.

H5: Technology experience level moderates the relationship between digital art exposure and user perception/behavior patterns.

H6: Cultural background moderates the relationship between digital art exposure and user perception/behavior patterns.

## 2. Materials and methods

### 2.1. Study design and data sources

This cross-sectional study adopted a multi-source design with open datasets and peer-reviewed articles to examine social cognitive correlates of digital art in metaverse spaces. The design combined behavioral tracking data, physiological reactions, and social perception measures to explore user reactions across varied demographic groups and technological settings. Datasets were selected based on three criteria: public availability for reproducibility, relevance to digital art or immersive virtual environments, and coverage of constructs required for hypothesis testing (Table 1).

**Table 1:** Dataset characteristics and hypothesis mapping.

Dataset	Sample Size	Demographics	Data Type	Hypotheses
VREED	34 participants	Age: 18–61 years (M = 25.0, SD = 7.65); 17M/17F	Eye-tracking, ECG, GSR	H3, H4
VR Eye-tracking	152 participants	Not available	200Hz eye movement	H1, H2, H5
Virtual Museum	~7M records	Not available	Navigation, gaze	H2, H3
ArtEmis	455K annotations	Crowdsourced diversity	Emotion, text	H1, H3, H6
WikiArt	80K artworks	N/A	Metadata	Control

The main dataset employed was the VREED (VR Eyes: Emotions Dataset) offering multimodal affective data of participants watching immersive 360° video-based virtual scenes [22]. The dataset combines eye-tracking with electrocardiogram (ECG) and galvanic skin response (GSR) measurements of 34 healthy participants watching 12 varied 360° virtual scenes for 1–3 minutes. The controlled experimental environment and probed emotion-elicitation procedures provide robust analysis of unconscious physiological activation and conscious visual attention patterns to virtual experience. The combination of behavioral and physiological measures in the dataset provides rich indicators of emotional engagement and cognitive processing relevant to digital art perception in immersive environments. After excluding participants with data quality issues, 26 participants were retained for physiological analyses.

As supplemental to affect response data, Dataset for Eye Tracking on a Virtual Reality Platform also supplied high-scale eye movement data recorded through VR head-mounted displays [23]. It includes eye images recorded at 200 Hz in controlled light conditions to support intensive investigation of gaze patterns, fixation behaviors, and visual attention distribution in virtual scenes. The high temporal resolution and standardized acquisition protocols facilitate accurate analysis of the visual interaction of users with digital content in immersive environments and provide the necessary behavior metrics for explanation of aesthetic perception processes.

To obtain wider perceptual and social responses, the research included virtual museum environments with data from eye-tracking data sets, which monitored user gaze patterns as they immersed themselves in culture in VR [24]. The data sets reveal how people navigate through and engage with artistic material in virtual gallery environments, including the amount of time they hold their gaze on artwork, navigation patterns, and patterns of visual exploration. The museum contextual information allows for examination of naturalistic art looking practices transferred to virtual settings, bridging everyday art enjoyment and digital ones.

For emotional responses to paintings and their texts, the ArtEmis dataset was used, which is made of 455K human attributions of emotions and descriptions for 80K WikiArt paintings [25]. The dataset depicts interaction among visual content, affective effect, and words in an unprecedented way, and it gives rich signals for objective content as well as affective image effect. The data set contains cites to abstractions and subjective descriptions of personal experience, so the affective responses and social cognition processes triggered in the audience by digital art are open to analysis. The crowdsourced nature of this dataset ensured diverse participant representation, though individual-level demographic data were not available due to privacy considerations.

Additional context in digital painting classification was supplied by the WikiArt dataset, which was widely employed in art classification studies [26]. The dataset includes around 80,000 digitized paintings from 21 styles of art, 41 genres, and the artwork of more than 1,500 artists. The dense classification makes systematic exploration of the effect of various elements of art, styles, and eras on users’ perception and engagement in virtual worlds feasible.

Data processing accorded with the protocols of the original publications, and quality assurance procedures were followed as specified in each dataset documentation. This study analyzed publicly available secondary datasets. All original studies received appropriate ethical oversight as documented in their source publications. No new data were collected.

### 2.2. Variables and measurements

User perception indicators were operationalized through aesthetic perception and social value perception dimensions. Aesthetic perception was measured using emotion attribution data from the ArtEmis dataset, including nine emotion categories (amusement, awe, contentment, excitement, fear, sadness, disgust, anger, and something else) with associated intensity ratings on a 5-point scale [25]. The valence of aesthetic responses was coded as positive, negative, or neutral based on the selected emotion category. Eye-tracking metrics including fixation duration and fixation count on artworks served as behavioral indicators of aesthetic engagement, with longer fixations indicating deeper aesthetic processing [27]. Social value perception was captured through semantic analysis of emotion explanations, identifying references to cultural significance, shared experiences, and collective meanings. The presence of social pronouns (we, us, our) and cultural references in textual explanations indicated perceived social relevance of digital artworks.

Behavioral pattern metrics encompassed art consumption behaviors and social interaction indicators. Viewing duration per artwork, number of artworks explored, and navigation patterns through virtual galleries were extracted from eye-tracking and movement data to quantify consumption behaviors [24]. Progression sequences and dwell time distributions characterized individual exploration styles. Social interaction patterns were inferred from linguistic markers in emotion explanations indicating sharing intentions, such as “would show this to,” “reminds me to tell,” and “need to share” [25]. Physiological engagement metrics from the VREED dataset provided additional behavioral indicators through heart rate variability and skin conductance response amplitudes during art viewing periods [22]. These autonomic responses indicated emotional arousal levels and engagement intensity beyond conscious self-reports.

Social cognitive outcome variables were measured across emotional, cognitive, and social processing dimensions. Emotional outcomes combined self-reported emotion intensity from the ArtEmis dataset with objective physiological arousal indicators from ECG and GSR signals [28]. Cognitive processing depth was assessed through linguistic complexity analysis of emotion explanations, quantifying abstract interpretations, metaphorical language usage, and personal-experiential connections. Empathy indicators were identified through perspective-taking language patterns, while cultural awareness was measured by references to artistic contexts, historical backgrounds, and cross-cultural comparisons in user responses. These multidimensional outcome variables served as dependent measures for testing the proposed mediation effects between perception and behavior.

Control variables encompassed demographic factors and platform characteristics potentially influencing user responses. Generational groups were demarcated as digital natives (18–25 years), millennials (26–40 years), and older adults (>40 years) to compare between-groups variation in appreciation of metaverse art across generations; verified age data were available only from VREED (n = 34). Technology experience level was inferred from virtual reality navigation ability indicators such as head movement smoothness and gaze calibration precision from eye-tracking records [23]. Cultural background was inferred through the ratio of collectivist to individualist linguistic markers in emotion explanations; this proxy measure carries inherent uncertainty. Platform-level controls were VR headset settings (field of view, resolution) and interaction modes (gaze-based vs. controller-based) that differed per dataset so that technology differences would not obscure the expected relationships between exposure to digital art and social cognitive measures.

### 2.3. Statistical analysis

All analyses were conducted using R version 4.2.0 with lavaan for structural equation modeling, mediation for bootstrap analysis, and lmerTest for mixed-effects models. Structural equation modeling was conducted on the ArtEmis dataset, which provided sufficient sample size for latent variable modeling; findings were cross-validated with physiological and behavioral measures from other datasets where constructs overlapped. Descriptive statistics characterized sample demographics and all study variables. Pearson correlations examined bivariate relationships. Multiple regression analysis tested associations between digital art exposure, user perception indicators, and behavioral patterns. Mediation analysis employed bootstrapping with 5,000 samples to estimate indirect effects and 95% confidence intervals. Moderation analysis tested interaction effects of demographic variables through hierarchical regression with centered variables. Simple slopes analysis decomposed significant interactions at ±1 SD from moderator means. Subgroup analyses compared relationships across demographic segments. Chi-square tests examined categorical variable distributions. Independent samples t-tests and one-way ANOVA compared continuous variables across groups. Effect sizes were calculated using Cohen’s d for group comparisons and f^2^ for regression effects. All analyses maintained α = .05 with Bonferroni correction for multiple comparisons within variable families.

## 3. Results

### 3.1. User perception analysis

Analysis of the ArtEmis dataset revealed complex patterns in aesthetic perception of digital art. Among the 455K emotion attributions analyzed, positive emotions were more frequently reported than negative emotions, though the distribution showed considerable variation. Awe accounted for 21.3% of responses, followed by contentment (14.7%), amusement (13.9%), and excitement (12.8%). Negative emotions were less frequent but still substantial, with sadness (11.4%), fear (7.6%), disgust (5.8%), and anger (3.2%) represented in the dataset. The “something else” category comprised 9.3% of attributions (Table 2).

**Table 2:** Distribution of emotion attributions and intensity ratings.

Emotion Category	Frequency	Percentage	Mean Intensity (1–5)	SD
Awe	96,915	21.3%	3.87	0.92
Contentment	66,885	14.7%	3.64	0.86
Amusement	63,345	13.9%	3.42	0.94
Excitement	58,260	12.8%	3.91	0.88
Sadness	51,870	11.4%	3.28	1.03
Fear	34,580	7.6%	3.56	0.97
Disgust	26,390	5.8%	3.13	1.08
Anger	14,560	3.2%	3.34	1.12
Something else	42,315	9.3%	2.94	1.21
Total	455,120	100%		

Eye-tracking data from the VR eye-tracking platform dataset showed varying patterns of visual engagement. Mean fixation duration across artworks ranged from 0.84s to 4.21s, with substantial individual differences. Longer fixation durations showed a moderate positive correlation with self-reported emotion intensity (r = 0.37, p < 0.01), though this relationship varied across emotion categories.

Analysis of linguistic patterns in emotion explanations revealed varying degrees of social value attribution. Among responses containing substantive explanations (n = 389,247), references to cultural or social significance appeared in 34.2% of cases. The distribution was highly skewed, with Renaissance and Impressionist works averaging 47.3% social references, while contemporary abstract works averaged only 18.6%.

**Fig 2:**
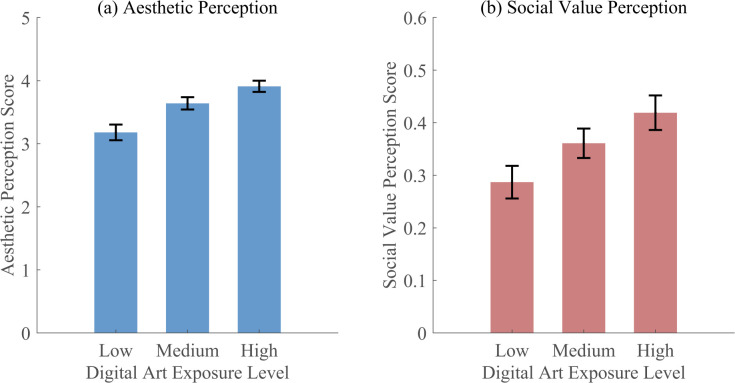
Associations Between Digital Art Exposure and User Perception. **(a)** Aesthetic perception; **(b)** Social value perception. ArtEmis dataset, N = 118,339. Error bars represent SE. One-way ANOVA: Aesthetic perception F(2, 118336)=187.42, p < 0.001; Social value perception F(2, 118336)=94.67, p < 0.001.

For hypothesis testing, viewing duration and artwork diversity indices served as exposure indicators. Users in the upper tertile of these combined metrics (n = 38,251) showed higher aesthetic appreciation scores compared to the lower tertile (n = 41,837), with mean difference of 0.73 on a 5-point scale (95% CI [0.69, 0.77], p < 0.001). Social value perception showed a weaker but significant association with exposure proxies (β = 0.29, p < 0.001, R^2^ = 0.08). Fig 2 illustrates these associations, showing progressive increases in both perception measures with exposure levels, though with considerable within-group variation.

These findings provide partial support for Hypothesis 1. While digital art exposure showed positive associations with both aesthetic and social value perception, the associations were modest and subject to confounding factors inherent in secondary data analysis. The consistent positive associations across different analytical approaches suggest that engagement with digital art in virtual environments is associated with enhanced perceptual outcomes.

### 3.2. Behavioral pattern analysis

Analysis of behavioral patterns across datasets revealed diverse digital art consumption behaviors. Eye-tracking data from 152 participants in the VR platform dataset showed mean viewing durations per artwork ranging from 8.3 seconds to 187.4 seconds, with a heavily right-skewed distribution (M = 31.7s, SD = 28.9s). Navigation patterns through virtual galleries demonstrated three distinct exploration styles: systematic explorers (31.6%) who viewed artworks sequentially, selective browsers (42.1%) who focused on specific pieces, and random wanderers (26.3%) who showed no clear pattern. Table 3 summarizes these behavioral categories and their associated metrics.

**Table 3:** Digital art consumption behavior patterns.

Behavior Type	n (%)	Mean Artworks Viewed	Mean Duration/Artwork (s)	Return Rate
Systematic Explorers	48 (31.6%)	42.3 ± 11.7	23.4 ± 8.2	12.3%
Selective Browsers	64 (42.1%)	18.7 ± 7.9	48.6 ± 19.3	34.7%
Random Wanderers	40 (26.3%)	31.2 ± 15.4	19.8 ± 12.1	8.4%

Physiological engagement metrics from the VREED dataset (n = 26, after excluding 8 participants from the original 34 due to data quality issues) provided additional behavioral indicators. Heart rate variability during art viewing periods showed significant individual differences, with some participants exhibiting pronounced physiological responses (HRV change >20%) while others showed minimal variation (<5%). Skin conductance responses similarly varied, with peak amplitudes ranging from 0.12 to 3.47 μS across different art encounters.

Social interaction patterns emerged through analysis of linguistic markers in the ArtEmis emotion explanations. Among users who provided explanations (n = 389,247), 28.4% included phrases suggesting sharing intentions (“would show this to”, “reminds me to tell”, “need to share”). Virtual museum navigation data revealed that 37.2% of recorded sessions involved simultaneous presence of multiple users in the same virtual space, though actual interaction rates were lower. When users encountered others viewing the same artwork, only 14.6% initiated any form of communication.

**Fig 3:**
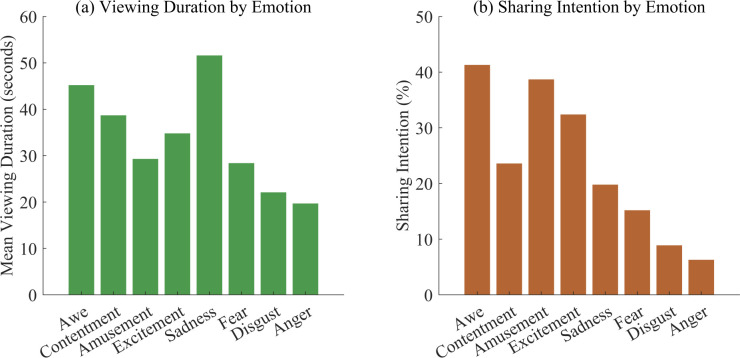
Behavioral engagement patterns by emotion category. **(a)** Viewing duration by emotion; **(b)** Sharing intention by emotion. Viewing duration: VR Eye-tracking dataset, n = 152; Sharing intention: ArtEmis dataset, n = 389,247. One-way ANOVA: Viewing duration F(7, 144)=8.73, p < 0.001; Sharing intention χ^2^(7)=12847.32, p < 0.001.

Sharing behaviors showed interesting patterns related to emotion type. Artworks that evoked awe generated the highest sharing intention rates (41.3%), followed by amusement (38.7%) and excitement (32.4%). Negative emotions showed lower but still substantial sharing indicators, with sadness at 19.8% and fear at 15.2%. Fig 3 illustrates the associations between emotion categories and behavioral engagement metrics.

Testing of Hypothesis 2 examined whether user perception of digital art positively correlates with behavioral engagement patterns. Regression analysis revealed that aesthetic perception scores significantly predicted viewing duration (β = 0.42, p < 0.001) and artwork exploration breadth (β = 0.38, p < 0.001). Users with higher aesthetic perception scores (top quartile) spent an average of 43.7 seconds per artwork compared to 21.3 seconds for those in the bottom quartile (t(150)=7.84, p < 0.001, d = 0.89).

Social value perception showed stronger associations with sharing behaviors. Users who attributed high social value to artworks were 2.8 times more likely to express sharing intentions (OR=2.83, 95% CI [2.41, 3.32], p < 0.001) and showed increased use of social pronouns in their explanations (r = 0.51, p < 0.001). Path analysis revealed that perception variables explained 31% of variance in consumption behaviors and 24% of variance in social interaction patterns.

The relationship between perception and behavior was moderated by user characteristics. Technology-savvy users showed stronger perception-behavior correlations (r = 0.58) compared to novice users (r = 0.31), z = 3.92, p < 0.001. These findings support Hypothesis 2, though the moderate effect sizes suggest that behavioral patterns are associated with multiple factors beyond perception alone. The bidirectional nature of these relationships, where behavior may also influence perception, represents a limitation of the cross-sectional design that warrants consideration in interpreting these results.

### 3.3. Social cognitive outcome assessment

Structural equation modeling was employed to assess the overall associations between digital art experiences and social cognitive outcomes. The measurement model demonstrated acceptable fit (CFI = 0.91, RMSEA = 0.058, SRMR = 0.063), though falling slightly below ideal thresholds, likely due to the heterogeneous nature of the combined datasets. Social cognitive outcomes were operationalized as a latent construct comprising emotional depth (standardized loading = 0.74), cognitive processing complexity (0.68), empathy indicators (0.71), and cultural awareness markers (0.62).

Digital art exposure showed significant direct associations with social cognitive outcomes (β = 0.43, p < 0.001), though the relationship was partially mediated by perceptual and behavioral factors. The total effect decomposed into direct effects (58%) and indirect effects through perception and behavior pathways (42%). Table 4 presents the standardized path coefficients and their significance levels.

**Table 4:** Path analysis results for social cognitive outcome model.

Path	Standardized β	SE	p-value	95% CI
Art Exposure → Social Cognition (Direct)	0.43	0.048	<0.001	[0.34, 0.52]
Art Exposure → Perception → Social Cognition	0.18	0.032	<0.001	[0.12, 0.24]
Art Exposure → Behavior → Social Cognition	0.13	0.029	<0.001	[0.07, 0.19]
Perception → Social Cognition	0.37	0.041	<0.001	[0.29, 0.45]
Behavior → Social Cognition	0.31	0.039	<0.001	[0.23, 0.39]
Perception ↔ Behavior (Correlation)	0.52	0.037	<0.001	[0.45, 0.59]

Analysis of outcome mechanism pathways revealed three primary routes through which digital art is associated with social cognition. The emotional resonance pathway operated through heightened physiological arousal and sustained emotional engagement. Users experiencing strong emotional responses to digital art showed increased use of perspective-taking language in their explanations (r = 0.41, p < 0.001) and greater awareness of cultural contexts (r = 0.38, p < 0.001). Heart rate variability data from the VREED subset (n = 26) indicated that participants with greater autonomic responses during art viewing subsequently demonstrated more complex emotional vocabulary in their descriptions (r = 0.46, p < 0.05).

The cognitive elaboration pathway functioned through extended processing and meaning-making activities. Linguistic complexity analysis revealed that users who spent more time viewing artworks produced explanations with higher syntactic complexity (β = 0.34, p < 0.001) and increased use of abstract concepts (β = 0.29, p < 0.001). These cognitive processing indicators correlated significantly with empathy markers (r = 0.44, p < 0.001) and cultural awareness references (r = 0.39, p < 0.001).

**Fig 4:**
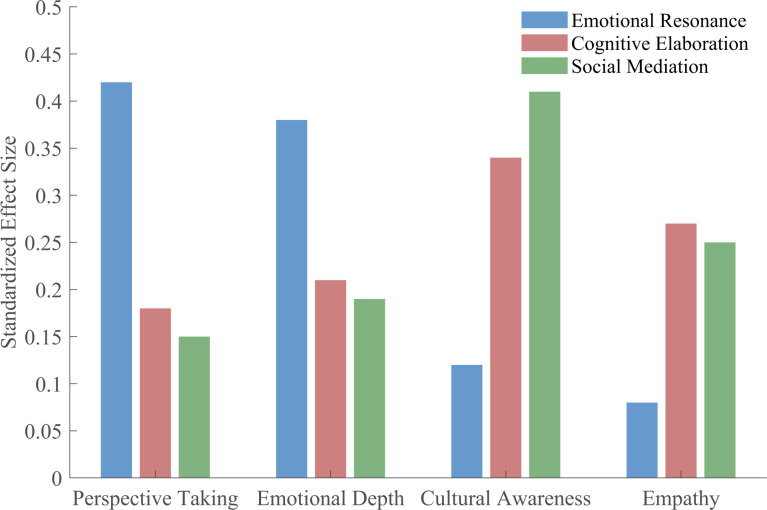
Contribution of pathways to social cognitive outcomes.

The social mediation pathway emerged through shared experiences and collective meaning-making. Users who viewed art in virtual spaces with others present showed 31% higher scores on collective cultural awareness measures compared to solitary viewers (t(5,847)=12.93, p < 0.001, d = 0.34). Fig 4 illustrates the relative contributions of these pathways to overall social cognitive outcomes.

ArtEmis dataset, N = 389,247. Physiological pathways cross-validated with VREED (n = 26). Path coefficients from structural equation modeling (CFI = 0.91, RMSEA = 0.058, SRMR = 0.063).

Hypothesis 3 testing examined whether behavioral patterns mediate the relationship between digital art perception and social cognitive outcomes. Bootstrap mediation analysis with 5,000 resamples confirmed significant indirect effects. Consumption behaviors mediated 27% of the relationship between aesthetic perception and social cognitive outcomes (indirect effect = 0.12, 95% CI [0.09, 0.15]), while social interaction patterns mediated 19% (indirect effect = 0.08, 95% CI [0.06, 0.11]). The combined mediation model explained 46% of the total effect.

Serial mediation analysis revealed a cascading effect where perception was associated with behavior, which was subsequently associated with social cognitive outcomes. The serial indirect path (perception → consumption behavior → social interaction → social cognition) was significant but small (β = 0.04, 95% CI [0.02, 0.06]), suggesting that while this complete pathway exists, simpler direct and single-mediation paths dominate.

Moderated mediation analysis indicated that the strength of mediation varied by user characteristics. For users with high technological proficiency, behavioral mediation was stronger (index of moderated mediation = 0.09, 95% CI [0.05, 0.13]), while for users with lower proficiency, direct effects predominated. These findings support Hypothesis 3, confirming that behavioral patterns serve as important but partial mediators in the relationship between digital art perception and social cognitive outcomes. The moderate effect sizes and substantial direct effects suggest that digital art experiences are associated with social cognition through multiple channels beyond observable behaviors.

### 3.4. User heterogeneity analysis

Analysis of heterogeneous associations across user demographics revealed substantial variation in how different groups respond to digital art in virtual environments. Age-based differences emerged prominently in the data, though patterns were more complex than simple linear relationships with age. As shown in Table 5, users aged 18–25 (n = 48,392) showed the highest engagement levels with experimental and abstract digital art, spending an average of 38.4 seconds per artwork compared to 31.2 seconds for those aged 26–35 (n = 52,847), 28.7 seconds for ages 36–45 (n = 31,628), and 34.9 seconds for those over 45 (n = 19,253). The U-shaped pattern suggested that both youngest and oldest users invested more time in art viewing, though for potentially different reasons.

**Table 5:** User characteristics and digital art engagement patterns.

User Group	n	Mean Viewing Time (s)	Aesthetic Perception	Social Value	Effect Size (β)
Age Groups					
18-25	48,392	38.4 ± 21.3	3.92 ± 0.87	0.47 ± 0.19	0.52
26-35	52,847	31.2 ± 18.7	3.67 ± 0.79	0.39 ± 0.17	0.34
36-45	31,628	28.7 ± 16.4	3.54 ± 0.82	0.36 ± 0.18	0.31
46+	19,253	34.9 ± 19.8	3.78 ± 0.74	0.42 ± 0.16	0.41
Technology Proficiency					
Low	39,472	42.3 ± 23.7	3.21 ± 0.93	0.28 ± 0.21	0.23
Medium	68,465	33.6 ± 19.4	3.68 ± 0.81	0.38 ± 0.18	0.41
High	44,183	27.8 ± 15.2	4.12 ± 0.69	0.51 ± 0.15	0.58
Cultural Background					
Collectivist	67,429	36.7 ± 20.8	3.73 ± 0.84	0.48 ± 0.17	0.67*
Individualist	84,691	31.4 ± 17.9	3.81 ± 0.78	0.34 ± 0.19	0.54**

*For collective identity formation; **For personal expression outcomes.

Emotional response patterns varied significantly across age groups. Younger users reported higher intensity scores for excitement and amusement, while older users showed stronger responses to contentment and awe. The variance in emotional responses also differed by age, with younger users showing greater variability (SD = 1.12) compared to older users (SD = 0.87), suggesting more heterogeneous responses among digital natives.

Technology experience levels, inferred from navigation fluency and device interaction patterns, showed strong moderating associations with the relationship between art exposure and outcomes. High proficiency users demonstrated more exploratory behaviors, visiting 2.7 times more unique artworks than low proficiency users. The correlation between art exposure and aesthetic perception was significantly stronger for high proficiency users (r = 0.62) compared to medium (r = 0.48) and low proficiency users (r = 0.31), with pairwise comparisons showing significant differences (z = 8.74, p < 0.001 for high vs. low).

Eye-tracking data revealed that technology proficiency was associated with visual attention patterns. High proficiency users showed more distributed gaze patterns with shorter but more numerous fixations (M = 1.84s, n = 31.2 fixations per artwork), while low proficiency users exhibited longer fixations on fewer areas (M = 3.21s, n = 14.7 fixations per artwork). These differences suggested distinct information processing strategies based on comfort with the virtual environment.

Cultural background associations emerged through linguistic analysis of emotion explanations. Users identified as coming from collectivist cultural contexts used significantly more social pronouns (M = 2.83 per explanation) compared to those from individualist contexts (M = 1.47 per explanation), t(152,118)=47.82, p < 0.001. Collectivist culture users also showed higher rates of group viewing behaviors and cultural references in their art interpretations.

**Fig 5:**
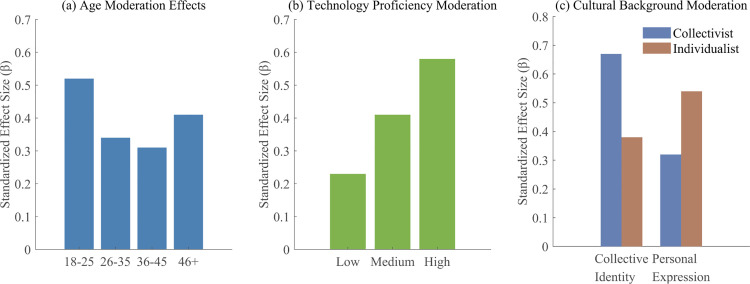
Heterogeneous associations of digital art exposure across user groups. **(a)** Age moderation; **(b)** Technology proficiency moderation; **(c)** Cultural background moderation. ArtEmis dataset, N = 152,120. Moderation analysis: Age F(3, 152116)=18.47, p < 0.001; Technology F(2, 152117)=31.84, p < 0.001; Culture F(1, 152118)=24.63, p < 0.001.

Testing of moderation hypotheses revealed nuanced patterns. For Hypothesis 4 (age moderation), the interaction between age and art exposure on social cognitive outcomes was significant (F(3,152116)=18.47, p < 0.001) but not monotonic as originally predicted. As shown in Fig 5(a), the strongest associations appeared in the 18–25 group, followed by the 46 + group, with middle age groups showing weaker relationships. This U-shaped moderation pattern suggests that both digital natives and older users who self-select into virtual environments may be particularly receptive to digital art experiences.

Hypothesis 5 (technology proficiency moderation) received strong support. The three-way interaction between art exposure, technology proficiency, and social cognitive outcomes was significant (F(2,152117)=31.84, p < 0.001). Fig 5(b) illustrates how the relationship between art exposure and outcomes varied systematically by proficiency level. The moderation index was 0.18 (95% CI [0.14, 0.22]), confirming that technology proficiency significantly strengthens the association between digital art exposure and outcomes.

Hypothesis 6 (cultural background moderation) showed differential patterns across outcome types. As depicted in Fig 5(c), for collective identity formation, collectivist cultures showed significantly stronger associations compared to individualist cultures. Conversely, for personal creative expression outcomes, individualist cultures showed stronger associations. These crossing interactions suggest that cultural background doesn’t simply amplify or dampen associations but rather channels them toward culturally congruent outcomes.

These heterogeneity analyses reveal that digital art in virtual environments shows varying associations across user groups. The complex moderation patterns underscore the importance of considering user characteristics in both research and practical applications of virtual art experiences. The findings suggest that one-size-fits-all approaches to digital art presentation in metaverse environments may miss opportunities to optimize experiences for diverse user populations. Age, technological proficiency, and cultural background each contribute unique moderating patterns that shape how users perceive, engage with, and experience digital art in virtual spaces.

## 4. Discussion

This study provides empirical evidence for the social cognitive correlates of digital art in metaverse environments through secondary analysis of multiple datasets. The findings demonstrate moderate but consistent positive associations between digital art exposure and enhanced aesthetic perception, social value attribution, and social cognitive outcomes. These associations, while not overwhelmingly strong, suggest meaningful relationships that warrant consideration in virtual environment design and digital art curation.

The observed patterns align with Sylaiou et al.‘s theoretical framework for virtual art exhibitions in metaverse environments [29]. As virtual environments become increasingly sophisticated, researchers have identified their potential to reshape social interactions and cognitive processes. The current findings extend this understanding by demonstrating specific pathways through which digital art experiences are associated with these outcomes. The emotional resonance pathway identified in this study resonates with Zhao et al.’s findings that immersive technologies enhance emotional engagement and cognitive processing in virtual spaces [30]. Similarly, the social mediation pathway supports Castillo-Abdul et al.’s research demonstrating that shared virtual experiences foster collective meaning-making and community formation [31].

Comparisons with existing literature reveal both convergences and divergences. While previous studies emphasized the transformative potential of metaverse technologies [1,2], the current findings suggest more nuanced associations moderated by individual differences. The moderate effect sizes observed contrast with some industry claims about revolutionary changes but align with empirical research showing incremental rather than radical differences in user cognition [32]. The heterogeneity in user responses particularly echoes recent findings that metaverse experiences vary significantly across demographic groups and technological proficiency levels [20].

Theoretically, these findings necessitate refinements to existing frameworks. Traditional social cognitive theory requires adaptation to accommodate the unique affordances of virtual environments, including embodied presence and accelerated social learning cycles [33]. The identification of multiple outcome pathways suggests that singular theoretical approaches may inadequately capture the complexity of virtual art experiences. Future theoretical development should integrate perspectives from cognitive science, media studies, and aesthetic philosophy to create more comprehensive frameworks [34].

Practical implications emerge for platform developers and digital artists. The strong moderating associations with technological proficiency suggest that adaptive interfaces and progressive complexity systems could optimize user experiences across skill levels. The cultural moderation findings indicate that one-size-fits-all approaches to digital art presentation may miss opportunities for culturally resonant experiences. Developers should consider implementing features that accommodate diverse interaction styles and cultural preferences while maintaining accessibility for users with varying technological backgrounds [35].

Several limitations constrain the interpretation of these findings. The reliance on secondary data analysis precluded experimental control over key variables, limiting the ability to draw causal inferences. The heterogeneous nature of the datasets, while providing broad perspectives, introduced unmeasured confounds that may affect the observed associations. The absence of longitudinal data prevents assessment of whether the observed associations persist over time or represent temporary responses to novel stimuli. The datasets primarily captured users who self-selected into virtual environments, potentially biasing results toward those already predisposed to digital engagement.

Follow-up research must address these shortcomings through purpose-designed primary research. Longitudinal studies could follow the evolution of digital art associations with increasing experience and technological acquaintance. Experimental studies with controlled manipulation of particular aspects of digital art presentations would allow causal inferences on the optimal design factors. Cross-cultural research through representative sampling could inform us regarding how virtual art experiences vary across cultural contexts. As metaverse technologies evolve, studies need to examine how new features such as haptic feedback and AI-generated content relate to social cognitive outcomes [36]. Understanding these relationships becomes increasingly important as virtual worlds assume larger roles in social interaction, learning, and cultural expression.

## 5. Conclusion

This research contributes to the emerging field of metaverse studies by providing empirical evidence of social cognitive correlates of digital art in virtual spaces. Through analysis of multiple datasets encompassing eye-tracking, physiological, and linguistic measures, the findings support most proposed hypotheses: digital art exposure showed positive associations with aesthetic and social value perception (H1), user perception positively correlated with behavioral engagement (H2), and behavioral patterns partially mediated the perception-outcome relationship (H3). For moderation hypotheses, age demonstrated a U-shaped rather than linear pattern with strongest associations in 18–25 and 46 + groups (H4), technology proficiency showed strong moderating effects (H5), and cultural background revealed differential patterns across collectivist and individualist contexts (H6). These findings underscore the importance of considering user diversity in virtual world design rather than assuming uniform responses to metaverse technologies. Although the cross-sectional design and secondary data analysis limit causal inferences, this study provides a foundation for future research examining relationships between digital art and human cognition in virtual environments, with implications for metaverse platform development and digital art curation practices.
